# A novel drug delivery system using acyclovir nanofiber patch for topical treatment of recurrent herpes labialis: A randomized clinical trial

**DOI:** 10.1002/cre2.512

**Published:** 2021-12-05

**Authors:** Zahra Golestannejad, Faezeh Khozeimeh, Mohammad Mehrasa, Shahla Mirzaeei, Dorna Sarfaraz

**Affiliations:** ^1^ Department of Oral Medicine, Dental Research Center, School of Dentistry Isfahan University of Medical Sciences Isfahan Iran; ^2^ Department of Biotechnology, School of Advanced Sciences and Technologies University of Isfahan Isfahan Iran; ^3^ Pharmaceutical Sciences Research Center, Health Institute Kermanshah University of Medical Sciences Kermanshah Iran; ^4^ Dental Students Research Committee, School of Dentistry Isfahan University of Medical Sciences Isfahan Iran

**Keywords:** acyclovir, herpes labialis, nanostructured drug delivery, patch

## Abstract

**Objectives:**

Topical treatment with acyclovir cream has shown low efficacy in recent studies. Nano drug delivery systems, have received much attention in recent decades. The aim of this study was to compare the efficacy of acyclovir nanofiber patch with acyclovir cream.

**Material and Methods:**

In this double‐blind three‐armed randomized clinical trial, a total of 60 patients with recurrent labial herpes, were randomly divided into three groups, each consisting of 20. The patients in the first, second, and third groups were treated with acyclovir nanofiber patch, placebo nanofiber patch, and acyclovir cream, respectively. A numerical scale was used by the patients to record the self‐reported symptoms. Symptoms score, crusting time and healing time were assessed by the clinician. Kruskal‐Wallis test was used to compare the symptoms between the three groups, a survival test was also performed to evaluate the crusting and healing time. Data were analyzed in SPSS V22 at *P*‐value < 0.05.

**Results:**

The mean scores of symptoms at baseline were 1.6, 1.5, and 1.4 in the first, second, and third groups, respectively. The symptoms were not significantly different between the three groups on different treatment days. The mean crusting time was 2.3, 2.4, and 2.6 days in the three groups, and the mean healing time was 7.4, 7.2, and 7.7 days, respectively. Crusting time and healing time were not significantly different between the three groups.

**Conclusions:**

Acyclovir nanofiber patches are recommended for accelerating symptom relief in recurrent labial herpes, however, they are not effective in shortening the crusting or healing time.

Clinical Trial Registration Number: IRCT20141124020073N2.

Registered in: Iranian Registry of Clinical Trials (www.irct.ir).

## 1 INTRODUCTION

Recurrent herpes labialis, also known as cold sore or fever blister, is a recurrent orofacial viral lesion, which is primarily caused by herpes simplex virus (HSV) type 1 (Spruance & Kriesel, 2002). Nucleoside analogues, such as acyclovir, are common drugs prescribed for the treatment of cold sore lesions (Monavari et al., 2014). These drugs inhibit viral DNA polymerase and act as chain terminators (Monavari et al., 2014). Topical treatment has shown several advantages, such as higher drug delivery to the tissue, accurate targeting of the lesion, and better acceptance among patients (Balfour Jr, 1999). However, topical treatment with acyclovir is limited due to its low transdermal permeability (penetration into the dermis) (Lembo et al., 2018) and poor solubility in water (Patel et al., 2018) which leads to low efficiency of drug delivery and absorption at site and therefor high doses of the cream are needed. Also, topical use of the cream has side effects, such as a burning or stinging sensation, itching, sensitivity, and flakiness of the skin (Lembo et al., 2018; Prajapati et al., 2017). Moreover, high doses of the cream can lead to allergies, uncontrolled drug release, and unpleasant odor and flavor (Lembo et al., 2018).

In recent years, various drug delivery methods have been developed for appropriate administration of acyclovir (Al‐Dhubiab et al., 2015; Bhupinder & Newton, 2016; Monavari et al., 2014; Vedula et al., 2016). The carrier in the drug delivery system is expected to have the following properties: lack of immunogenicity, reproducibility, biocompatibility, acceptable biodegradation time, non‐toxicity, and continuous activation to reach the target (De Koker et al., 2012). Nano drug delivery systems improve drug release and stability by maintaining the drug concentration and overcoming biological barriers to cellular uptake (J.M. Rabanel et al., 2012). Nanofibers, which are among drug carriers in nano drug delivery systems, have received much attention in recent decades. This group of drugs consists of natural and/or synthetic polymers with dimensions of 20 to 900 nm (Ansari et al., 2012).

Nanofibers have advantages, such as high surface‐to‐volume ratio, controlled drug release, and increased drug penetration into biological barriers, such as the epithelium, which enhances drug delivery to the lesion site (Ansari et al., 2012; Yoo et al., 2009). Increased biological accessibility and performance improvement reduce the need for higher doses of the drug, and therefore, decrease toxicity and side‐effects (Shirwaikar et al., 2008). Polyvinyl alcohol (PVA) is one of the synthetic polymers, approved by the Food and Drug Administration (FDA) as a drug carrier (Choi et al., 2008; Shalumon et al., 2011).

So far, there have been no clinical trials comparing acyclovir cream with acyclovir nanofiber. Considering the poor water solubility and low transdermal permeability of acyclovir cream, besides the potential of nanofiber patches in the improvement of biological accessibility, the aim of this study was to compare two drug formulations (acyclovir nanofiber patch and acyclovir cream) in the treatment of recurrent herpes labialis.

## 2 MATERIALS AND METHODS

In this double‐blind three‐armed randomized clinical trial, we recruited a total of 60 patients with recurrent labial herpes, who were referred to the Department of Oral Medicine of Isfahan University of Medical Sciences, Isfahan, Iran in 2020. Lesions were diagnosed by an oral medicine specialist. The inclusion criteria were as follows: 1) recurrent (secondary) not primary lesions, 2) lesions in the vesicular stage (size ≤1 cm). On the other hand, the exclusion criteria were as follows: 1) any allergy to acyclovir or the history of severe allergy to other drugs, 2) diagnosis of systemic diseases; liver or kidney disorders, 3) inherited or acquired immunodeficiency disease, 4) manipulations, such as scratching or shaving at the site, 5) use of birth control pills, pregnancy, or lactation, 6) use of antiviral, anti‐inflammatory, steroidal, or analgesic drugs, 7) use of local agents, such as cosmetic products, lip lubricants, and sunscreen at the site of the lesion during treatment. The study objectives were explained to the patients and written informed consents were obtained.

### 2.1 Randomization process

The patients were randomly allocated to one of two intervention groups (acyclovir nanofiber patches, acyclovir cream) or the control group (placebo nanofiber patches) each of 20. Considering small recruited sample size and for overall balance reasoning between the three study arms, we randomized the recruited patients employing permuted block randomization method with a block size of 3.

The first group received a package containing 15 acyclovir nanofiber patches, the second group received a package containing 15 placebo nanofiber patches, and the third group received a 5% acyclovir cream tube (Iran Daru, Tehran, Iran). Considering the double‐blind design of this study, the patients were not informed about the content of the packages. The clinicians were also unaware of the content of packages provided for the first and second groups (acyclovir and placebo nanofiber patches, respectively), but they were informed about the cream. The patients were instructed to use the prescribed drugs at the site of the lesion three times a day (after a meal) for 5 days.

The aim of this study was to measure the symptoms (pain, itching, and burning sensation) at the lesion site and to evaluate the crusting time (from baseline until the formation of crust) and healing time (from baseline until complete disappearance of crust) of lesions following the use of each pharmaceutical formulation. To determine the degree of symptoms, the patients in each group were evaluated daily using a numerical scale, ranging from 0 to 10 from the beginning of treatment (baseline) until the symptoms disappeared. To measure the crusting time and healing time, the patients were followed‐up on the second, fourth, and sixth days from the baseline for clinical examinations.

### 2.2 Design, formulation, and evaluation of acyclovir nanofiber patches

For this purpose, 1 g of polyvinyl alcohol (PVA) (99% hydrolyzed, average *M*_w_ = 89–98 kDa) (Simaab Novin Azmaparseh, Tehran, Iran) was dissolved in 10 ml of deionized water at 80°C for 12 h and stored for 1 day. Next, 100 mg of acyclovir powder (Merck, Germany) was dissolved in 10 ml of PVA solution and stirred with a magnetic stirrer for 5 h to produce a homogeneous solution. The prepared solution was treated in the electrospinning process (FNM Nanotechnology Co., Tehran, Iran). The electrospinning parameters were as follows: voltage of 25 kV, flow rate of 0.4 ml/h, and a 15‐cm distance between the needle and the collector. To evaluate the size, distribution, and structure of acyclovir nanofiber patches, scanning electron microscopy (SEM) was used. The SEM images represented the surface structure.

### 2.3 Design and formulation of placebo nanofiber patches

For this purpose, 1 g of PVA (Simaab Novin Azmaparseh, Tehran, Iran) was dissolved in 10 ml of deionized water at 80°C for 12 h and stored for 1 day. The prepared solution was then treated in an electrospinning process (FNM Nanotechnology Co., Tehran, Iran). The electrospinning parameters were as follows: voltage of 25 kV, flow rate of 0.4 ml/h, and a 15‐cm distance between the needle and the collector. To evaluate the size, distribution, and structure of nanofiber patches, SEM was performed.

### 2.4 Statistical analysis

Data (symptoms score, crusting time, and healing time) are presented as mean ± SD. Kruskal‐Wallis test was performed to compare the mean scores of symptoms on each treatment day between the three experimental groups. Survival test was also performed for crusting time and healing time, and the results were compared between the three groups, using log‐rank test. The level of significance was set at 0.05 in each statistical analysis. Data were analyzed in SPSS version 22.

## 3 RESULTS

**Figure 1:**
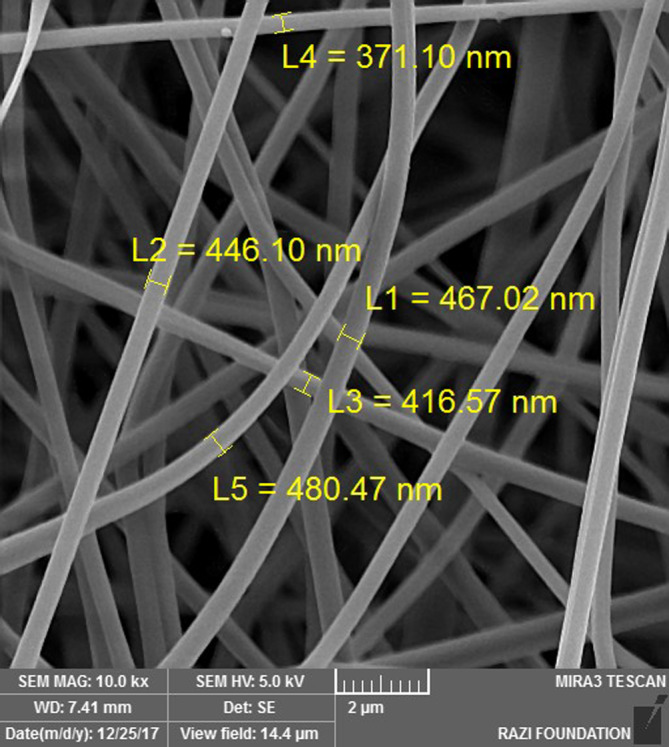
SEM micrograph of the electrospun placebo nanofibers (electrospinning parameters 25 kV, 15 cm, and 0.4 ml h^−1^)

**Figure 2:**
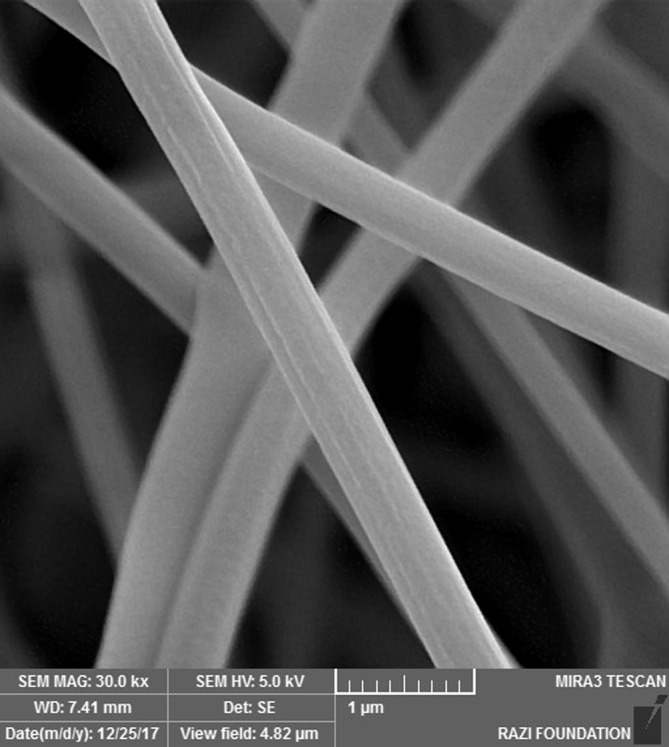
SEM micrograph of the electrospun acyclovir nanofibers (electrospinning parameters 25 kV, 15 cm, and 0.4 ml h^−1^)

The SEM image of the ribbed nanofiber structure showed that both acyclovir nanofiber patch and placebo nanofiber patch were made successfully. The surface and internal structure of the acyclovir nanofiber patch were identical. The images showed that drug molecules were uniformly loaded and distributed on the surface and between the fibers. The approximate size of nanofibers was estimated at 436 ± 39 nm (Figures 1 and 2).

A total of 49 patients completed the study, and 11 patients were treated with the replacement method. The mean scores of symptoms at baseline were 1.6, 1.5, and 1.4 in the first (acyclovir nanofiber patch), second (placebo nanofiber patch), and third (acyclovir cream) groups, respectively. By the fourth day, all of the symptoms had improved in the three groups. The mean scores of the symptoms on each treatment day, according to the prescribed drug, are shown in Table 1. Kruskal‐Wallis test was also performed to compare the mean scores of symptoms on each treatment day between the three groups. The results showed no significant difference in the symptoms on different treatment days between the three groups (P‐values are shown in Table 1).

**Table 1:** The mean and standard deviation (SD) of symptom scores on different treatment days in the three experimental groups

Type of drug	Baseline	First day	Second day	Third day	Fourth day
	Mean (SD)	Mean (SD)	Mean (SD)	Mean (SD)	Mean (SD)
Acyclovir nanofiber patch	1.6 (0.7)	1 (0.85)	0.3 (0.6)	0.1 (0.3)	0
Placebo nanofiber patch	1.5 (0.6)	1.2 (0.8)	0.5 (0.5)	0.1 (0.4)	0
Acyclovir cream	1.4 (0.6)	1.2 (0.7)	0.5 (0.8)	0.1 (0.4)	0
*P*‐value	0.622	0.745	0.45	0.56	–

**Figure 3:**
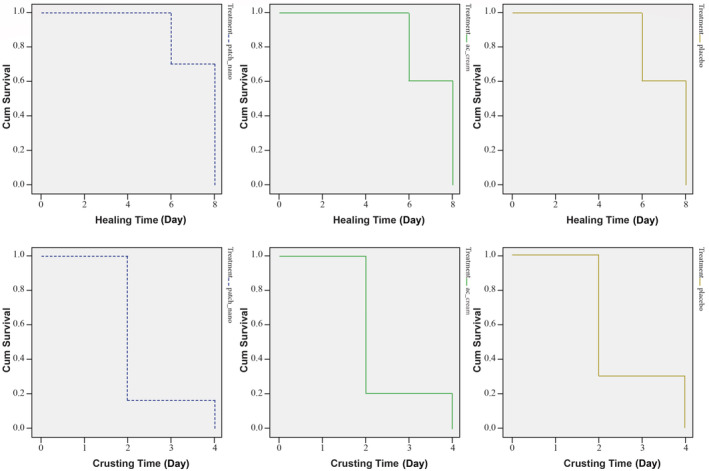
Survival curves of crusting time and healing time in the three experimental groups

The mean crusting time was 2.3, 2.4, and 2.6 days in the first, second, and third groups, respectively. Based on the results of log‐rank test, crusting time was not significantly different between the three groups (Figure 3) (*P* = 0.509). Moreover, the survival curves indicated that the mean healing time was 7.4, 7.7, and 7.2 days in the first, second, and third groups, respectively. Based on the log‐rank test, healing time was not significantly different between the three groups (Figure 3) (*P* = 0.754).

## 4 DISCUSSION

The present findings showed that on different treatment days, the symptoms were not significantly different between the groups. Previous studies have shown that viral replication and viral load are associated with the severity of recurrent HSV attacks. The severity of these attacks is measured with regard to the symptoms (Lembo et al., 2013). Since local antiviral drugs have clear and direct effects on viral replication (Spruance & Crumpacker, 1982), they alleviate the symptoms by reducing viral replication. However, topical treatment with acyclovir cream is limited due to the very low solubility of acyclovir in water, its low transdermal permeability, and consequently, limited penetration into the dermis. In contrast, in nanofiber patch formulations, drug particles are used in mixture with polymer matrix (Polyvinyl alcohol), which are highly soluble in water. These nanofibers carry drug particles and increase the hemogenisity of drug in water, resulting in a significant increase in drug bioavailability. Also, nanofiber systems can offer greater drug loading than many other types of drug delivery systems (Chew et al., 2005; Megelski et al., 2002).

Unlike previous reports, the results of the present study revealed that two different formulations of acyclovir did not influence symptom relief in comparison with the placebo and were not significantly different from each other (Arduino & Porter, 2006; Leflore et al., 2000; Spruance et al., 2002). Since there is no clinical study comparing acyclovir nanofiber with routine acyclovir cream formulations, the results of the present study are not comparable with similar research. Nevertheless, different in vitro studies have shown that the acyclovir nanocarrier formulation is more effective than its routine formulation, which is in contrast with the present study.

In this regard, Monavari et al. (2014) reported the higher antiviral activity of nano‐niosome‐based acyclovir in vitro. Moreover, Al‐Dhubiab et al. (2015) investigated the drug delivery of buccal films impregnated with acyclovir‐loaded nanospheres in three in vitro and ex vivo conditions. The in vitro results showed the potential of the film for controlled drug release over a long period, and the ex vivo results indicated controlled penetration, longer absorption of acyclovir, and increased bioavailability of the film. Vedula et al. (2016) also reported the delivery of higher drug concentrations, using carboxymethyl cellulose acetate butyrate nanoparticles containing acyclovir.

Furthermore, Bhupinder and Newton (2016) reported the higher encapsulation efficiency, drug loading capacity, and drug release of acyclovir solid lipid nanoparticles (SLNs) as a skin drug delivery system in vitro. Prajapati et al. (2017) found greater skin penetration and drug stability using acyclovir‐loaded nanogel, compared to acyclovir cream on the rat skin. Moreover, EL‐Assal (2017) investigated the skin drug delivery of acyclovir‐loaded SLNs versus acyclovir cream in a rat model. The in vitro results showed controlled drug release and drug stability of SLNs, and the ex vivo results showed greater drug permeation and accumulation in the rat skin, compared to the routine acyclovir cream.

Furthermore, Patel et al. (2018) reported that in vitro drug release of acyclovir nano‐emulsion gel was significantly higher than that of the pure drug. Donalisio et al. (2018) also showed the increased skin penetration and antiviral activity of acyclovir‐loaded chitosan nanospheres, compared to the free drug. The possible cause of discrepancy between the present results and the mentioned studies is the capacity of the examined nanostructures to increase drug release and stabilize drug concentration against biological barriers in the cellular uptake process. On the other hand, nanofiber patches evaluated in the present study had limited advantages, such as high surface‐to‐volume ratio and controlled drug release, and failed to improve drug penetration into the biological barrier (epithelium), which leads to an enhancement in drug delivery to the lesion site.

In nanofiber patches, drug particles are used in mixture with nanosized fibers (nanofibers), which are highly soluble in water. These nanofibers carry drug particles and increase their solubility in water. Nanofiber systems can lead to higher drug loading, compared to many other drug delivery systems and have no mass transfer constraints seen in other polymeric drug delivery systems (Chew et al., 2005; Megelski et al., 2002). Electrospinning has emerged as a major technology for the manufacture of produced nanofibers. These structures have filament diameters and morphologies comparable to those found in the extracellular matrix of human tissues. Therefore, nanofiber structures can improve the adaptation processes in the tissue and provide a large surface area for the nanofiber network; therefore, they can be applied as a local drug delivery system (Ignatious & Sun, 2006; Rahaman et al., 2007; Verreck et al., 2003).

The present results showed that crusting time and healing time in the acyclovir nanofiber patch group was not significantly different from the groups using acyclovir cream and placebo nanofiber patches. In other words, the acyclovir nanofiber patch and routine acyclovir formulation did not have any significant effects on the healing or crusting time of herpes lesions. Despite the clear effect of local antiviral drugs on the severity of recurrent herpes labialis attacks, previous studies have shown that these drugs affect the attack duration in very limited cases (10%–15%) (Harmenberg et al., 2010; Spruance & Kriesel, 2002); therefore, they do not have a clear effect on the healing time. The results of the present study, consistent with previous research, showed that local antiviral acyclovir, both in routine and nanofiber patch formulations, had no significant effects on the healing time or crusting time, compared to the placebo. This finding is possibly related to the progress of recurrent herpes lesions. In these lesions, viral replication in epithelial cells results in the death of infected cells and triggers inflammatory activities following cell death.

The inflammatory effects are triggered within the first 8 h of lesion formation and ultimately result in a wound, which then enters the recovery phase. Since inflammatory cascades are mostly activated within the first 8 h of the emergence of labial herpes lesions, and local antiviral drugs cannot inhibit the activated inflammatory process due to lack of anti‐inflammatory properties, these drugs are not effective in the attack duration, and recurrent herpes labialis follows its natural course (i.e., papule, vesicle, ulcer, and ultimately healing) (Hull et al., 2014). In addition, comparison of the formation technique between the two acyclovir formulations (cream and nanofiber patch) showed that a lower amount of drug is utilized in the structure of nanofibers, compared to the cream, which leads to better patient acceptance and lower expenses. It should be noted that formation of nanofibers requires complicated methods and special conditions.

The limitations of the present study include failure to compare the acyclovir patch with acyclovir cream in terms of the release profile, penetration percentage, and absorption capacity in vitro. The number of the participants was also limited.

The acyclovir nanofiber patch is recommended for accelerating symptom relief. However, it is not recommended for shortening the crusting time or healing time in recurrent labial herpes.

## CONFLICT OF INTEREST

The authors declare that they have no conflict of interest.

## AUTHOR CONTRIBUTIONS

All authors contributed to the study conception and design. Research design and material preparation was performed by Zahra Golestannejad and Faezeh Khozeimeh, Data acquisition and analysis, interpretation and preparation of the manuscript was performed by Dorna Sarfaraz, Mohammad Mehrasa, and Shahla Mirzaeei. All the authors read and approved the final manuscript.

## Data Availability

The data that support the findings of this study are available on request from the corresponding author. The data are not publicly available due to privacy or ethical restrictions.
